# Clinical Evaluation of Pediatric Pulse Oximeters in South Africa: Protocol for a Cluster Randomized Controlled Trial

**DOI:** 10.2196/82888

**Published:** 2026-03-30

**Authors:** Holly B Schuh, Marieke Van der Zalm, Margaret Van Niekerk, Lise-Marie Laubscher, Abenathi Mcinziba, Steve Gomas, Sunaina Kapoor, Shubhada Hooli, Lario Viljoen, Andre Gie, Bareng A S Nonyane, Pierre Goussard, Anneke C Hesseling, Carina King, Eric D McCollum

**Affiliations:** 1 Department of Epidemiology, Bloomberg School of Public Health, Johns Hopkins University, Baltimore, MD, United States; 2 Global Program in Pediatric Respiratory Sciences, Eudowood Division of Pediatric Respiratory Sciences, Department of Pediatrics, Johns Hopkins School of Medicine, Rubenstein Building, 200 North Wolfe Street, Baltimore, MD, United States, 1-410-955-2035; 3 Desmond Tutu TB Centre, Department of Paediatrics and Child Health, Stellenbosch University, Clinical Building, Francie Van Zijl Dr., Parow Valley, Cape Town, South Africa, 27 021-938-9062; 4 Springer Design, Danville, CA, United States; 5 Division of Pediatric Emergency Medicine, Department of Pediatrics, Baylor College of Medicine, Houston, TX, United States; 6 Department of Paediatrics and Child Health, Faculty of Medicine and Health Sciences, Stellenbosch University, Cape Town, South Africa; 7 Department of International Health, Bloomberg School of Public Health, Johns Hopkins University, Baltimore, MD, United States; 8 Department of Global Public Health, Karolinska Institutet, Stockholm, Sweden

**Keywords:** respiratory tract infections, pneumonia, hypoxemia, child, infant, pulse oximetry, health facilities, low- and middle-income countries, clinical decision-making

## Abstract

**Background:**

The burden of children with lower respiratory infections and low blood oxygen levels (hypoxemia) is high, and outcomes are poor in low- and middle-income countries (LMICs). Pulse oximeters noninvasively measure the capillary oxyhemoglobin saturation (SpO_2_) to identify hypoxemia, but high-quality devices designed for the unique needs of children are rarely available in primary health care clinics (PHCs) in LMICs, where children initially access care.

**Objective:**

This study aims to evaluate whether 2 pediatric pulse oximeters co-designed with health care workers (HCWs) in LMICs improve the correct SpO_2_ management of children in PHCs compared to a standard pulse oximeter.

**Methods:**

We are conducting a pragmatic 3-arm cluster randomized controlled trial in the Eastern Khayelitsha, Northern, and Tygerberg areas of Cape Town, South Africa, over 18 months between 2024 and 2026. We plan to enroll 1200 children aged younger than 2 years with an acute respiratory infection from 18 PHCs randomized to implement one of 3 pulse oximeters, either 1 standard-of-care device or 2 intervention devices. HCWs in selected PHCs will administer the intervention. Our primary outcome will be “correct SpO_2_ management,” an intermediate clinical end point between device implementation and hypoxemia outcome, defined by the following three elements necessary to reduce inappropriately treated hypoxemia: (1) device adoption—HCW use of the device as evidenced by a HCW-documented SpO_2_ and pulse rate; (2) quality SpO_2_ measurement—SpO_2_ confirmed by reference device measurement within 2% SpO_2_ above or below the HCW-measured SpO_2_; and (3) correct SpO_2_ decision-making—an appropriate referral recommendation by the HCW. A concurrent mixed methods process evaluation will explore how, why, for whom, and to what extent these devices impact the clinical management of hypoxemic children. The primary analysis will be intention-to-treat. For all primary and secondary outcomes, we will conduct pairwise comparisons between the 2 intervention arms and the control arm.

**Results:**

Data collection commenced in 2024, and results are expected from 2026 to 2027. As of December 2025, enrollment has been completed in 12 clinics.

**Conclusions:**

While there are notable challenges inherent in designing a trial to evaluate whether pulse oximeters improve HCW SpO_2_ management of children at PHCs, our protocol development process attempted to address all potential limitations and sources of bias to maximize the trial’s future impact.

## Introduction

Lower respiratory infections, including pneumonia, remain the leading infectious cause of death globally for children aged younger than 5 years [1,2]. Most lower respiratory infection–related deaths occur in low- and middle-income countries (LMICs), with inequitable distribution both between and within countries [1,3,4]. Key quality-of-care implementation gaps have hampered the effectiveness of the World Health Organization’s (WHO) Integrated Management of Childhood Illnesses (IMCI) guidelines [5], which are commonly used for pediatric pneumonia case management in LMICs. Interventions to improve the identification of severely ill children within the IMCI approach could improve outcomes, with routine use of pulse oximetry, to noninvasively measure blood oxygen levels via the capillary oxyhemoglobin saturation (SpO_2_), providing one such opportunity [6,7].

Hypoxemia, defined as a low SpO_2_, is both common in children with pneumonia, estimated at 28% within the African context and 23% in outpatient settings [8], and is associated with increased mortality [9]. Therefore, effectively identifying these children early in their care-seeking pathway is fundamental to reducing mortality in high-burden, low-resource contexts. While IMCI recommends SpO_2_ measurements for children with suspected pneumonia, pulse oximetry devices for measuring SpO_2_ are not widely implemented in primary health care clinics (PHCs) in LMICs, where most children first access care [6]. Referral decisions are therefore largely based on subjective clinical danger signs, which do not accurately identify hypoxemia [10,11]. Routine care is ineffective at identifying treatment failure in nonsevere pneumonia patients sent home with first-line antibiotics, and little evidence exists on its burden [12-14]. Estimating the prevalence of treatment failure among children with pneumonia at PHCs would fill a key evidence gap on outcomes and resources needed in this setting.

A challenge in the implementation of pediatric pulse oximetry is that devices are largely designed for surgical and inpatient continuous monitoring in high-resource and adult populations. The use case for outpatient pediatric patients seen in low-resource settings differs considerably from adults—ease of placement, robustness, battery life, motion artifact and blood perfusion variability, and smaller surface areas for measurement are important and can pose greater difficulty [15]. To date, there have been a limited number of efforts to design pulse oximeter devices specifically for this target group, meaning that many devices currently used in pediatric outpatients are not fit for purpose. Devices that have been promoted for use on infants and children are either too costly for scale-up in LMICs, as they are dependent on expensive and often fragile specialized probes, or their performance has not been validated in the pediatric population, leading to performance issues when implemented in real-world contexts.

The Phefumla (meaning “breathe” in isiXhosa) trial will evaluate the clinical impact of 2 novel pediatric pulse oximeters, both co-designed with HCWs and specifically for children presenting to high-burden and low-resource outpatient settings, on the quality of care for children with acute respiratory infections (ARIs) in Cape Town, South Africa. Both devices were designed to address known issues with pediatric oximetry measurements related to poor probe fit onto the digits of infants and children and related performance issues, but with different approaches. Specifically, one device is an investigational probe-less reflectance oximeter designed to obtain measurements on digits, hands, feet, and the forehead [16]. The second device is a regulatory-approved transmissive oximeter clip probe uniquely designed to fit on the big toe and across the foot of infants and younger children and on the fingers of older children [17]. In transmissive oximetry, light passes through tissue to a detector located on the opposite side of the body, while in reflectance oximetry, light is emitted and detected on the same side of the body [18]. This trial aims to understand not just the impact of these pediatric pulse oximeters on clinical outcomes but also how these devices perform and are implemented within routine care, when compared to standard pulse oximeter devices in PHCs in the Western Cape of South Africa.

## Methods

### Study Design

**Figure 1.**
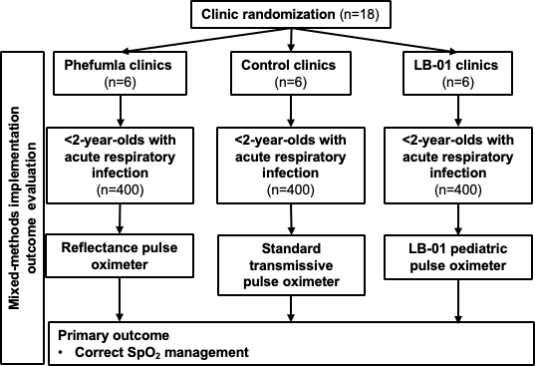
Study design. SpO_2_: capillary oxyhemoglobin saturation.

The Phefumla trial is a pragmatic 3-arm cluster randomized controlled trial consistent with elements of a type 1 hybrid effectiveness-implementation design, given that the primary end point (“correct SpO_2_ management”) is a composite intermediate clinical outcome (see the Trial Outcomes section) [19]. The trial is conducted in the Eastern Khayelitsha, Northern, and Tygerberg areas of Cape Town, South Africa, over an 18-month period between 2024 and 2026 (Figure 1). A total of 18 clusters, defined as PHCs, will be randomized to the Phefumla device [16] (prototype version 1.1; Springer Designs), the LB-01 device [17] (Acare), or standard care, with the primary outcome being “correct SpO_2_ management” among children aged younger than 2 years with ARI (Textbox 1). Implementation outcomes will be defined according to the “reach, effectiveness, adoption, implementation, and maintenance” (RE-AIM) framework [19,20] and will be used to explore how, why, for whom, and to what extent these novel pediatric pulse oximetry devices impact the clinical management of hypoxemic children.

Textbox 1. **Primary trial research question and hypotheses.**

Research question: Do pediatric pulse oximeters designed for low-resource contexts improve capillary oxyhemoglobin saturation management in primary health care clinics (PHCs) compared to standard care?

Hypothesis 1: Health care workers (HCWs) using the LB-01 oximeter will correctly manage a higher proportion of children aged 0 to <24 months at PHCs with acute respiratory infection (ARI) than with the current standard pulse oximeter.

Hypothesis 2: HCWs using the prototype Phefumla oximeter will correctly manage a higher proportion of children aged 0 to <24 months at PHCs with ARI than with the current standard pulse oximeter.

The evaluation was designed in line with the RE-AIM framework [20]. Effectiveness will be assessed among children aged younger than 2 years through a prospective cohort of children presenting to study clinics. Reach, adoption, and implementation will be assessed through the mixed method process evaluation, involving facility audits, qualitative observations, and health care worker skills assessments. Maintenance of HCW pulse oximeter practice will be assessed through a follow-up facility visit 6 months after patient recruitment is completed.

We will also nest a descriptive prospective cohort study throughout the trial period to evaluate clinical outcomes for recruited children, pooling 3 trial arms and following them for 2 weeks after enrollment. The primary outcome of this nested substudy will be 2-week postenrollment treatment failure.

### Setting

The trial will sample PHCs from the Eastern Khayelitsha (6 PHCs), Tygerberg (6 PHCs), and Northern (6 PHCs) health subdistricts. Khayelitsha has an official population of 450,000 and is 90.5% Black African [21], although researchers and organizations working there estimate the population to be closer to 1 million [22]. Khayelitsha has a young population, with more than 40% of its residents aged younger than 19 years, residing in informal housing with high caregiver unemployment and limited access to running water. HIV and tuberculosis prevalence are high, with maternal HIV prevalence being 29.5%—the highest in the Western Cape Province— and the tuberculosis incidence of 1389 per 100,000 population exceeded the national average of 834 per 100,000 in 2017 [23]. The National Department of Health of South Africa uses IMCI guidelines for the management of children at PHCs.

### PHC Eligibility and Recruitment

Eligible PHCs are defined as public facilities with no known planned closures during the study period, that provide health care to children aged 0 to less than 24 months, and are based in the Eastern Khayelitsha, Tygerberg, or Northern health subdistricts of the Western Cape. A list of eligible facilities will be obtained from the Western Cape Department of Health and City of Cape Town, and this will act as the sampling frame for clusters. Through discussion with Western Cape Department of Health and the City of Cape Town officials, we purposively selected the 18 study PHCs. The intervention will be delivered at a facility level, with all HCWs of any cadre currently employed (either on a temporary or permanent basis) who provide care for children aged younger than 2 years being eligible for participation. Activities will occur through sequential site enrollment (see also Randomization and Blinding and Impact Evaluation Procedures sections).

### Intervention and Control

We will be comparing the Phefumla (arm 1) and LB-01 (arm 2) devices to pulse oximeter devices currently used in routine care (arm 3 control). The intervention devices were both developed through a human-centered design approach with HCWs working in LMIC contexts and are not currently routinely used in the trial setting. The transmissive (ie, measuring SpO_2_ by shining light *through* tissue to the opposite side of the body) LB-01 device is Conformité Européenne (CE) marked and commercially available [17]. The reflectance (ie, measuring SpO_2_ by detecting light *reflected* from vessels below the skin on the same side of the body). Phefumla device is an investigational device that passed International Organization for Standardization (ISO) 80601-2-61:2017 regulatory testing for accuracy and safety and is eligible for CE marking and US Food and Drug Administration clearance but cannot be used for clinical decision-making during routine care [16]. The reference device (Rad-G; Masimo), a transmissive oximeter device, will be used across all 3 trial arms to ensure participant safety and comply with nonsignificant risk study standards. A summary of the technical specifications of the trial devices is provided in Table 1.

**Table 1.** Technical specifications of trial pulse oximeters.

Characteristic	Arm 1	Arm 2	Arm 3^a^	Reference
Device	Phefumla pulse oximeter	AH-MX LB-01 pulse oximeter	CONTEC CMS5100 patient monitor	Masimo Rad-G pulse oximeter
Oximeter type	Handheld	Handheld	Desktop	Handheld
Regulatory markings	ISO^b^ 80601-2-61:2017 (passed), investigational device	ISO 80601-2-61:2017; CE^c^ IIb classification	ISO 80601-2-61:2017; US FDA^d^ clearance	ISO 80601-2-61:2017; US FDA clearance, CE IIb classification
Patient population	Neonate, pediatric, and adult	Neonate, pediatric, and adult	Neonate, pediatric, and adult	Neonate and pediatric
Recommended measurement location	Skin surface	Big toe, finger, or thumb	Big toe, index finger, or thumb	Big toe, index finger, or thumb

a If a control facility has a different device, the health care workers will continue to use their standard device. If a control facility has no functional oximeter device, the CONTEC pulse oximeter will be provided.

b ISO: International Organization for Standardization.

c CE: Conformité Européenne.

d US FDA: US Food and Drug Administration.

All facility staff who routinely provide care for children aged younger than 5 years, regardless of cadre and trial arm, will be trained in pulse oximetry. Control facilities will use the Contec Medical Systems (CONTEC) pulse oximeter (Table 1). If a control facility has a different device, the HCWs will continue to use their standard device. If a control facility has no functional oximeter device, the CONTEC pulse oximeter will be provided. All intervention components are summarized in Table 2.

**Table 2.** Summary of intervention components across the 3 study arms.

	Arm 1	Arm 2	Arm 3
Pulse oximeter provision^a^	Phefumla device	LB-01	Existing device in facility^a^
Baseline training and pilot by study physician	Days 1-2 or 3 of enrollment period	Days 1-2 or 3 of enrollment period	Days 1-2 or 3 of enrollment period
Intervention enrollment period	Days 2 or 3 of enrollment period until 4-6 wk enrollment with a target sample size of 66-67 children	Days 2 or 3 of enrollment period until 4-6 wk enrollment with a target sample size of 66-67 children	Days 2 or 3 of enrollment period until 4-6 wk enrollment with a target sample size of 66-67 children
Direct observation(s) by study physician	After at least 3 wk of enrollment	After at least 3 wk of enrollment	After at least 3 wk of enrollment

a If the clinic does not have a functional oximeter, they are provided with a CONTEC device.

### Participants

Our primary target population is children aged 0 to less than 24 months. Eligibility criteria for trial recruitment include children aged 0 to less 24 months, routinely presenting to care with HCW-observed and/or caregiver-reported history of either cough and/or difficult breathing (ie, ARI), being a resident of the Western Cape, having a primary caregiver who agrees to provide contact details for follow-up and is able and willing to provide written informed consent. Children with any of the following WHO-defined emergency signs will be excluded: obstructed or absent breathing, severe respiratory distress (ie, grunting), signs of uncompensated shock (capillary refill time >3 s, elevated heart rate with weak pulse, and low or unmeasurable systolic blood pressure), coma, active convulsions, or diarrhea with severe dehydration (altered mental status, sunken eyes, very slow return after skin pinch, or any 2 of these).

If there is no viable site for measurement (eg, the participant is missing digits or has severe burns), this will be classified as an “unsuccessful measurement.” The inclusion criteria for ARI were chosen to reflect the WHO IMCI guidelines [5], as these signs are the entry point into an HCW conducting a full respiratory examination.

### Randomization and Blinding

Clinics will be randomized in a 1:1:1 ratio to intervention arm 1 (Phefumla device: 6/18, 33%), intervention arm 2 (LB-01 device: 6/18, 33%), and control (6/18, 33%). The 18 study clinics will be equally distributed across the 3 subdistricts (Khayelitsha Eastern, Northern, and Tygerberg) and were randomized using stratified randomization to give 2 clinics in each arm in each subdistrict. Using the same random number, these facilities were then put into blocks, with 1 clinic from each arm in each block (ie, 6 blocks of 3 clinics each). Within these blocks, a new random number was generated to assign the clinic recruitment order. Randomization will be done using Stata SE version 17 (StataCorp) [24].

Owing to the nature of the intervention, the only group masked to trial arm allocation is the trial statistician (BAN), who will conduct the primary trial analysis. All investigators will also be masked during the analysis phase of the trial. The key listing of PHCs by trial arm will be held by the trial epidemiologist (CK) and shared after the primary analysis results have been presented to a study advisory group comprising health professionals and researchers responsible for providing independent advising to the project.

PHC staff, caregivers, participants, and study staff at participating facilities cannot be blinded to the type of pulse oximeter. Caregivers at the PHC will be informed that there is an ongoing study. HCWs and eligible participants will be provided with information, but they will not know the trial arm of the PHC. HCWs and facility managers will be provided with information on the study design, research questions, procedures, and randomization. However, these individuals will not be informed of the primary outcome definition, nor which device is the control device.

### Impact Evaluation Procedures

Two study teams comprised 1 research nurse and 1 research counselor who will be supervised by 1 study physician and will conduct child and caregiver recruitment and follow-up. Study staff will be trained on the study protocol, data collection tools, ethics and informed consent, and SpO_2_ measurements. This will be followed by observed mock interviews, with a formal assessment of SpO_2_ measurement and interpretation skills. Study clinics will be enrolled sequentially, with participant recruitment occurring in 1 to 2 clinics simultaneously. At each clinic, participation will entail HCW training for the first 1 to 2 days of the enrollment period, followed by 1 to 2 days of piloting. At HCW trainings, study staff will review IMCI guidelines that include guidance on pulse oximetry use and demonstrate and train HCWs on the use of the assigned pulse oximeter devices. Study staff at control facilities will receive the same training but with a focus on the commercially available device available at the facility. Study staff will recruit 4 to 5 d/wk during clinic operating hours, for a 4‐ to 6-week period, depending on patient volume.

Study staff will follow a standard operating procedure for recruitment. Children will be screened, identified as eligible, managed routinely by clinic staff, selected to participate, and then enrolled by study staff. Informed consent will be provided in the caregiver’s preferred language of isiXhosa, English, or Afrikaans by trained study staff. After a child is triaged and completes their SpO_2_ evaluation by the HCW, study staff will collect 2 SpO_2_ measurements. The first SpO_2_ measurement will be done using the reference device, followed by the study arm device, with both initiated within 10 minutes of the routine HCW assessment. Following the final clinical assessment by the registered nurse or attending HCW, additional sociodemographic, vaccination, and contact information will be collected, and a clinical examination will be performed by study staff. If the reference SpO_2_ measurement clinically differs from the HCW’s measurement and may impact onward care, then it will be shared with the HCW to support their clinical decision-making (eg, the reference device and HCW measurement differ by being either above or below the 90% threshold used for oxygen initiation and onward referral). If an SpO_2_ reference measurement cannot be initiated within 10 minutes of the HCW measurement, the study data collector will identify this as a failed measurement, although all children will still have a reference SpO_2_ measurement obtained. Participant flow through the study will depend on patient flow in the PHC, but in all cases, a valid study measurement must be initiated within 10 minutes of the HCW measurement. In control clinics that are not using the CONTEC device, study staff will still use the CONTEC device for reference measurements. To optimize both participant safety and the timing of study SpO_2_ measurements, a delayed consent waiver is available for study staff to use in PHCs that more systematically obtain SpO_2_ measures during patient triage. Finally, trial pulse oximeters will be removed after each clinic’s implementation period, and clinics will be provided with a reference device for continued use for patient care.

### Trial Outcomes

The primary trial outcome is “correct SpO_2_ management” [15], a composite intermediate clinical end point between correct pulse oximeter use and hypoxemia outcomes, and is defined by the three elements necessary to reduce inappropriately treated hypoxemia: (1) device adoption—HCW use of the device as evidenced by an HCW-documented SpO_2_ and heart rate measured in room air (ie, off supplemental oxygen); (2) quality SpO_2_ measurement—a study staff–measured SpO_2_ with the reference device that is within a 2% SpO_2_ range above or below the HCW-documented SpO_2_; and (3) correct SpO_2_ decision-making—an appropriate referral recommendation provided by the HCW according to the WHO-defined hypoxemia definition (SpO_2_ <90%). A ±2% SpO_2_ range was selected, as it is comparable to the standard acceptable difference between the reference arterial blood gas oxyhemoglobin saturation and SpO_2_ measurements used in pulse oximeter development and testing and is the performance range stipulated in pulse oximeter manufacturer product specifications. A ±2% SpO_2_ range also applies to individuals with darker skin tones more common in LMIC settings [25] (see Table 3 for key terms). The secondary outcomes are correct SpO_2_ management according to varied definitions, quality measurement frequency, referral acceptance, and oxygen treatment.

**Table 3.** Key terms.

Term	Definition
ARI^a^	Reported or observed cough or difficulty breathing.
Pneumonia	ARI and fast breathing for age, chest wall indrawing, a general danger sign, or hypoxemia [5].
WHO^b^-defined hypoxemia	SpO_2_^c^ <90% while breathing room air [5].
Study-defined hypoxemia	SpO_2_ <94% while breathing room air [26].
Moderate hypoxemia	SpO_2_ 90%-93% while breathing room air.
Biologically plausible	SpO_2_ measurements achieving a stable plethysmography waveform with stable SpO_2_ (±1%) for 3-5 s and a heart rate between 1st and 99th centile for age [27].
Appropriate referral	Children with a SpO_2_ <90% were counseled to go to the hospital, given a prereferral dose of antibiotic according to IMCI^d^, and received supplemental oxygen (if available). Children with an SpO_2_ >90% may be referred due to other clinical signs—this will not be considered inappropriate.
Difficult breathing	Any abnormal breathing pattern reported by caregiver or observed by a HCW^e^ or study staff.
Fast breathing for age	Respiratory rate >60 breaths per minute if 0 to <2 mo of age, >50 breaths if 2 to <12 mo of age, or >40 breaths if 12 to <24 mo of age [5].
Chest wall indrawing	Bilateral inward pulling of the anterior chest wall subcostal tissue and lower ribs during inspiration [5].
General danger signs (2- to <24-mo-olds)	Vomiting everything, lethargy or unconscious, unable to drink or breastfeed, convulsions, or stridor at rest [5].
Neonatal danger signs (0- to <2-m-olds)	Unable to feed well, not moving at all or moves only when stimulated, severe chest indrawing, or grunting.
Unsuccessful SpO_2_ measurement	Study staff measurements initiated within 10 min of the HCW measurement using the device assigned to arm and reference device both indicate performance is not within ±2%.

a ARI: acute respiratory infection.

b WHO: World Health Organization.

c SpO_2_: capillary oxyhemoglobin saturation.

d IMCI: Integrated Management of Childhood Illnesses.

e HCW: health care worker.

### Power and Sample Size

The trial is powered to detect a difference in the primary outcome of “correct SpO_2_ management” in a pairwise analysis between each intervention arm and the control arm. We estimate that 55% of children within control clinics will meet the definition for correct SpO_2_ management [28]. Using the following parameters, we will be able to detect a 22% increase in the primary outcome: 18 months recruitment, 6 clusters per arm, 60 children per cluster (targeting 67 and allowing for ~10% attrition, ie, child enrolled but post-HCW visit examination incomplete), 2.5% alpha after applying Bonferroni correction [29], greater than 80% power, intraclass correlation coefficient of 0.05, and a coefficient of variation of cluster size of 0.2. This will give us a sample of 360 children per arm (N=1080). We will aim to recruit an average of 3 to 4 children each day from each facility, yielding approximately 1200 children.

### Statistical Analysis

The primary analysis will be intention-to-treat comparing correct SpO_2_ management between each intervention arm and the control arm, using a mixed-effects logistic regression model. We will assess the variability in cluster sizes and whether these are associated with the outcome and, if so, will use independence estimating equations with a logit link. As we anticipate a low level of missing data, our primary analysis will be a complete case analysis. The detailed statistical analysis plan is provided in Multimedia Appendix 1.

### Mixed Method Process Evaluation

The mixed method process evaluation will be conducted throughout the trial enrollment period, with quantitative data collected from all facilities, and ethnographic observations conducted in a subsample of 6 facilities (2 per arm). The process evaluation will aim to explore the contexts that shape HCW willingness to use pulse oximetry, how facility structures and patient pathways influence pulse oximetry uptake, HCW perceptions and preference for different devices and their usability, and the association of pulse oximetry use with referral acceptance. Process indicators organized according to the RE-AIM framework [20] are summarized in Table 4. Information on the wider context in which the intervention is being run will be documented through study team meeting notes, including health system disruptions (eg, staff strikes) and political actions (eg, the launch of new policies and elections).

**Table 4.** Summary process evaluation indicators and data sources.

RE-AIM^a^ domain	Definition	Indicator
Reach	The absolute number, proportion, and representativeness of children who are willing to participate in the study	Consent rate (data source: recruitment log; perspective: caregivers) Comparison of participants to nonparticipants (data source: recruitment log; perspective: caregivers) Socioeconomic profile of participants (data source: enrollment survey; perspective: caregivers) SpO_2_^b^ measurement acceptance (data source: enrollment survey; perspective: caregivers) Referral acceptance (data source: enrollment survey; perspective: caregivers)
Adoption	The absolute number, proportion, and representativeness of facilities and HCWs^c^ who are willing to participate in the study	Characteristics of PHCs^d^ (data source: baseline facility audit; perspective: PHCs) Availability of functional pediatric pulse oximeters at baseline (data source: baseline facility audit; perspective: PHCs) Characteristics of HCWs who participate (data source: baseline HCW survey; perspective: HCWs)
Implementation	At the facility level, fidelity to the intervention protocol, including consistency of delivery	HCW knowledge of pulse oximetry (data source: baseline HCW survey; perspective: HCWs) HCW proficiency in pulse oximetry (data source: HCW skills test; perspective: HCWs) Time to successful SpO_2_ measurement (data source: HCW skills test; perspective: HCWs) Perception of HCWs about the pulse oximeter (data source: HCW FDG^e^; perspective: HCWs) Perception of the HCWs about the mode of pulse oximeter implementation (data source: HCW FDG^e^; perspective: HCWs) Adaptation to the pulse oximeter implementation approach (data source: baseline facility audit and observations; perspective: PHCs)
Maintenance	The extent to which the use of pulse oximetry becomes institutionalized, measured 6 mo after intervention completion	HCW knowledge after 6 mo (data source: follow-up HCW survey; perspective: HCWs) HCW proficiency after 6 mo (data source: Follow-up HCW skills test; perspective: HCWs) Availability of functional pediatric pulse oximeters after 6 mo (data source: check-in logs; perspective: PHCs)

a RE-AIM: reach, effectiveness, adoption, implementation, and maintenance.

b SpO_2_: peripheral blood oxygen saturation.

c HCW: health care worker.

d PHC: primary health care clinic.

e FDG: focus group discussion.

Each PHC will have a baseline facility audit conducted after the PHC has provided consent and before any other trial intervention activities take place. The audit will collect data on clinical services, infrastructure, and human resources and will include inspections of oxygen and pulse oximetry equipment. A subset of 6 purposefully selected PHC will have 1 to 2 days of ethnographic observations, following a structured observation checklist, to explore pulse oximetry practice and patient pathways. For this subset of facilities, observations will be repeated during weeks 1 and 4 of trial recruitment and then again at the 6-month visit.

HCW surveys will be conducted with all HCWs before they take part in the baseline training to assess demographics and work history, knowledge of the IMCI guidelines and pulse oximetry, and training history. This survey will be repeated at the end of the clinic recruitment period (between 4 and 6 wk later), and again at the 6-month visit. HCWs will also be asked to do a pulse oximetry skills test at these 2 time points, during which they will conduct 2 measurements on different children, while being observed by study staff [17].

After the end of trial recruitment across all facilities, a discussion with HCWs from the study clinics will be held. We will invite 1 HCW from each of the study facilities to attend a meeting where we will present the key trial findings and facilitate a discussion of the results. We will aim for a mix of HCWs to ensure a range of trial perspectives and experiences.

### Embedded Prospective Cohort

The embedded prospective cohort comprised the same children enrolled in the trial. The objectives of the prospective cohort analyses are to determine the outpatient burden of treatment failure and hypoxemia in PHCs and to describe risks for treatment failure, hypoxemia, and mortality among children presenting with ARIs. Treatment failure will be defined as the proportion of enrolled children who reported that they were still sick or whose caregiver reported any of the following signs: cough at 14 days, difficulty breathing at 14 days, change in antibiotic treatment, readmission to the clinic or hospital, or death any time at or before 14 days after study enrollment, as confirmed via phone or by home visit.

### Ethical Considerations

Our protocol has been approved by the Human Research Ethics Committee of Stellenbosch University in South Africa (M23/08/024) and the Johns Hopkins School of Medicine Institutional Review Board (IRB00444460) for ethical research conduct and the protection of human subjects. Study nurses and coordinators will obtain informed consent in a private area of the clinic separate from the public waiting area. HCW and mother and child participation are voluntary, and participants will be informed of their ability to opt out. Participants will be compensated as required by the South African Health Products Regulatory Authority for time, inconvenience, and expense. Data collected will be manually entered into a secure, password-protected, encrypted tablet and will be downloaded daily with the REDcap (Research Electronic Data Capture) data management system hosted on a dedicated and secure server at Stellenbosch University. All audio recordings and transcriptions will be held on password-protected and encrypted study laptops, and access to data on REDcap will be restricted to only key personnel. Linking files for personal identifiers will only be accessible to the study principal investigator and key personnel. We will disseminate findings at international research conferences and in peer-reviewed journals.

## Results

The study was funded in 2023, with data collection commencing in 2024. As of December 2025, we have completed enrollment in 12 clinics. Trial results are expected in 2026 and will be disseminated through academic publications, conferences, and a stakeholder meeting with HCWs from the trial PHCs.

## Discussion

### Anticipated Findings

In this trial, we will evaluate the correct SpO_2_ management of two pediatric pulse oximeters: the CE-marked LB-01 device and the first prototype of the investigational Phefumla device, both of which have undergone extensive development and validation using an end user–informed, participatory, human-centered oximeter design process with HCWs in Malawi, Bangladesh, the United Kingdom, and South Africa [16]. Both devices were designed to address common barriers that limit the use of pulse oximetry for young children in low-resource settings, including lack of devices designed for pediatric patients, challenges with children moving during measurement, prohibitive device cost, unavailability of devices at facilities, and lack of health care provider awareness, training, and supervision [10,15,16,30-34]. During the development phase of both devices, we learned that important features of durability, battery life, and usability with children are essential to providers in low-resource contexts. We intend to compare correct SpO_2_ management—a composite intermediate clinical end point between correct pulse oximeter use and hypoxemia outcome—between these devices and other standard-of-care devices on the market to understand whether their design features favorably support effective implementation in real-world settings and what improvements can be incorporated into future versions to address any identified performance issues.

### Strengths and Limitations

Our study design and implementation have challenges. First, there is difficulty in not disrupting clinical care while still generating meaningful comparison measurements to evaluate real-world clinical decision-making. To lessen any disruption, we include a delayed consent option to permit study staff to obtain SpO_2_ measures immediately before HCWs in clinics that more rapidly do pulse oximeter testing during patient triage at the PHC. Second, the Phefumla device cannot be used for clinical decision-making in this study as it is an investigational device. This project did not have the implementation time frame and resources to seek the regulatory approvals required to conduct a significant risk study. To address this, we pair reference and HCW device measurements to augment participant safety. Third, to address any bias on our primary outcome of “correct SpO_2_ management” that may be introduced by the presence of study staff at clinics (Hawthorne effect), we conduct study device measurements in a separate setting to avoid observation of our staff measurements. Fourth, as SpO_2_ is not a static measurement, there is the possibility of measurement bias from sequential HCW and study staff SpO_2_ measurements. To mitigate this potential issue, we set a 10-minute time limit for when the study measurements must be initiated following HCW SpO_2_ measurement. While preparatory work informed our decision to include the CONTEC control device, we cater our training to allow control clinics to continue to use whatever device is available at the PHC, even if different. We prioritized pragmatism and the implementation focus of our study and expect minimal effect on device measurement differences. Collectively, these necessary but challenging aspects of our protocol require patient flow maps specific to each participating clinic to maximize opportunities for recruitment, consenting, and study measurement procedures without disrupting patient services. We expect that findings from our study will be generalizable to low-resource settings globally as well as areas of high disease burden.

In sum, although the importance of SpO_2_ monitoring is well accepted, with calls to make SpO_2_ a vital sign [35], translating this into practice has proven challenging. Research gaps remain around optimizing implementation and sustaining best practice, with 7 of the top 20 pediatric pneumonia research priorities [36] centered around pulse oximetry and oxygen adoption and health system impacts. This trial will fill some of these gaps in evidence.

## Supplementary material

10.2196/82888 Multimedia Appendix 1 Statistical analysis plan.

## Abbreviations

ARI: acute respiratory infection
CE: Conformité Européenne
CONTEC: Contec Medical Systems
HCW: health care worker
IMCI: Integrated Management of Childhood Illnesses
ISO: International Organization for Standardization
LMIC: low- and middle-income country
PHC: primary health care clinic
REDCap: Research Electronic Data Capture
SpO2: oxyhemoglobin saturation
WHO: World Health Organization
