# Supplementary material for: Clinical Evaluation of Pediatric Pulse Oximeters in South Africa: Protocol for a Cluster Randomized Controlled Trial
Source: JMIR Res Protoc. 2026 Mar 30;15:e82888. doi: 10.2196/82888 (PMC13035077; doi:10.2196/82888)
Supplement: Multimedia Appendix 1 [file resprot-v15-e82888-s001.docx]

**Statistical Analysis Plan**

**Version 1.1**

**Project Title: Pulse oximeter clinical evaluation**

**Principle Investigator:** Eric D. McCollum, Johns Hopkins University, USA

**Co-investigators:** Anneke C. Hesseling, Stellenbosch University, South Africa

Marieke van der Zalm, Stellenbosch University, South Africa

Carina King, Karolinska Institute, Sweden

Lario Viljoen, Stellenbosch University, South Africa

Lise-Marie Laubscher, Stellenbosch University, South Africa

Holly B. Schuh, Johns Hopkins University, USA

Bareng Aletta Nonyane, Johns Hopkins University, USA

**Funding Body:** Thrasher Research Fund (USA), NIH (USA)

**This SAP was prepared by**

Bareng Aletta Nonyane, Johns Hopkins University, USA

Carina King, Karolinska Institute, Sweden

Holly Shuh, Johns Hopkins University, USA

**SAP** **Version History:**

| Version 1.0 | 28^th^ July 2025  Original version by BAN, CN, HS |
| --- | --- |
| Version 1.1 | 11^th^ January 2026  Edited EDM, CN |

Table of Contents

[1. Purpose 3](#_Toc198629784)

[2. Study Aim and Objectives 3](#_Toc198629785)

[3. Study Design 4](#_Toc198629786)

[Aim 1: Methods, procedures and outcomes 4](#_Toc198629787)

[Participants 4](#_Toc198629788)

[Intervention and control arms 4](#_Toc198629789)

[Randomization and blinding 5](#_Toc198629790)

[Power and sample size 5](#_Toc198629791)

[Primary outcome 5](#_Toc198629792)

[Secondary outcomes 6](#_Toc198629793)

[Aim 2: Methods, procedures and outcomes 7](#_Toc198629794)

[Participants 7](#_Toc198629795)

[Power and sample size 7](#_Toc198629796)

[Primary outcomes 7](#_Toc198629797)

[Secondary outcomes 7](#_Toc198629798)

[4. Statistical analyses 7](#_Toc198629799)

[Aim 1: General Statistical Analysis Principles 7](#_Toc198629800)

[Masking: 7](#_Toc198629801)

[Analysis population: 8](#_Toc198629802)

[Target primary estimand 8](#_Toc198629803)

[Covariate adjustment to improve precision in the estimates 8](#_Toc198629804)

[Interim analyses 8](#_Toc198629805)

[Missing data 8](#_Toc198629806)

[Hypothesis tests and type 1 error rate 8](#_Toc198629807)

[Software 8](#_Toc198629808)

[Aim 1: Outcome definitions and analytic approaches 8](#_Toc198629809)

[Aim 2: General Statistical Analysis Principles 10](#_Toc198629810)

[Missing data 10](#_Toc198629811)

[Hypothesis tests and type 1 error rate 10](#_Toc198629812)

[Software 10](#_Toc198629813)

[Aim 2: Outcome definitions and analytic approaches 10](#_Toc198629814)

# Purpose

This is a primary Statistical Analysis Plan (SAP) for the Phefumla cluster randomized controlled trial (cRCT). This describes all statistical methods that are related to the primary and secondary outcomes from Aim 1 and Aim 2. The analysis plan for evaluating implementation outcomes will be given in a separate document.

# Study Aim and Objectives

The primary aim of this trial is to assess the impact and implementation of two novel pulse oximeters, compared to the current standard, in the context of paediatric outpatient care in Cape Town, South Africa. This aim will be addressed through a pragmatic 3-arm cluster randomised controlled trial consistent with elements of a Type 1 Hybrid Effectiveness-Implementation design given the primary endpoint (‘correct SpO_2_ management’) is a composite intermediate clinical outcome, with concurrent implementation outcome evaluations that will explore how, why, for whom, and to what extent can paediatric pulse oximetry devices improve the clinical management of hypoxemic children. We will embed a prospective cohort study, to describe survival and clinical outcomes for hypoxaemic children to provide critical contextual data needed for subsequent implementation at scale. This will be done with two inter-linked aims:

**Aim 1:** Determine whether paediatric pulse oximeters designed specifically for low-resource contexts (Acare LB-01 and Phefumla devices) improve *correct management of oxygen saturation* in primary health care (PHC) facilities in Cape Town, South Africa, when compared to current standard care. We hypothesize the following:

*Hypothesis 1.* Healthcare workers (HCWs) using the *Acare LB-01 oximeter* will correctly manage a higher proportion of children 0 to <24 months-of-age at PHCs with acute respiratory infections than with the current standard pulse oximeter.

*Hypothesis 2.* HCWs using the *Phefumla oximeter* will correctly manage a higher proportion of children 0 to <24 months-of-age at PHCs with acute respiratory infections than with the current standard pulse oximeter.

**Aim 2:** Determine the outpatient burden of treatment failure in PHCs in Cape Town, South Africa, and describe risks for treatment failure, hypoxemia, and mortality amongst children presenting with acute respiratory infections in a low-resource setting in Cape Town. The specific objectives include:

- Determine the prevalence of treatment failure at two weeks post-enrolment and describe the association between treatment failure and risk factors amongst children presenting with acute respiratory infections to PHCS in Khayelitsha, Cape Town.
- Estimate the period prevalence of hypoxaemia, defined as either a peripheral capillary oxyhemoglobin saturation (SpO_2_) <90%, 90-93%, or <94%, amongst children presenting with acute respiratory infections to PHCS in Khayelitsha, Cape Town.
- Describe demographic and clinical factors associated with SpO_2_ <90%, 90-93%, or <94% amongst children presenting with acute respiratory infections to PHCS in Khayelitsha, Cape Town.
- Determine the prevalence of mortality at two weeks post-enrolment and describe the association between mortality and risk factors amongst children presenting with acute respiratory infections to PHCS in Khayelitsha, Cape Town.

# Study Design

## Aim 1: Methods, procedures and outcomes

Aim 1 will be addressed through a pragmatic 3-arm cRCT conducted over an 18-month period (Figure 1). Clusters are defined as 18 primary clinics (PHCs) – 6 in each arm. The primary outcome is ‘*correct SpO_2_ management*’ (see *Primary Outcome* section for definition) among <2-year-olds with an acute respiratory infection on the day of recruitment. Secondary impact outcomes focus on different definitions of SpO_2_ management and HCW treatment decisions.

The trial overall will use the RE-AIM framework to guide implementation outcome evaluation, measuring outcomes and indicators to address all elements of Reach, Effectiveness, Adoption, Implementation and Maintenance.

**Figure 1.**


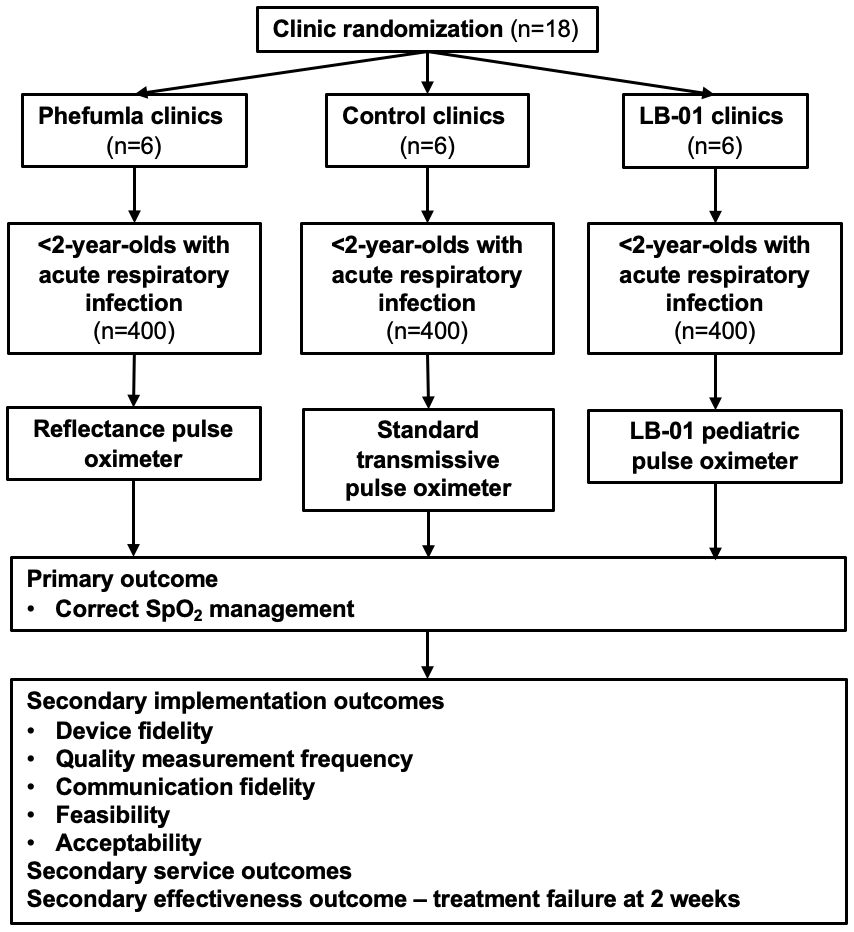


### Participants

Our primary target population for primary and secondary impact outcomes are children aged 0 to <24 months, who present with cough and/or difficult breathing. HCWs working in eligible and selected PHCs are the target for delivery of the intervention. The study inclusion and exclusion criteria are the same for Aims 1 and 2 and are fully detailed in the Protocol.

### Intervention and control arms

All facility staff who routinely provide care for children under-five years, regardless of cadre, within study clinics will be trained in the application of pulse oximeters on children, following a Standard Operating Procedure documented in Phefumla_SpO2MeasurementSOP_v2.1. The trial arms will differ only in terms of the pulse oximeter device that they are trained to use, with control facilities provided with the commercially available CONTEC^TM^ pulse oximeter that is routinely used at PHCs. One intervention arm will be provided with the Phefumla device, and one intervention arm will be provided with the LB-01 device. Intervention components are summarised in Table 3 of the Protocol.

### *Randomization and blinding*

The randomisation was conducted using Stata SE17 by Carina King, Karolinska Institutet, on the 12^th of^ August 2024, and documented with the .do file PhefumlaRandomisation_081224.do. The randomisation file was shared with Eric McCollum, Lise-Marie Laubscher and Margaret Van Nierkerk.

A list of 18 clinics, 6 in each of three Zones (Khayelitsha Eastern, Northern and Tygerberg), were first randomised using stratified randomisation to give 2 clinics in each arm in each Zone. Using the same random number, these facilities were then put into blocks – with one clinic from each arm in each block (i.e. 6 blocks of 3 clinics each). Within these blocks, a new random number was generated, using a random number generator. They were then ordered by this number, to assign the order in which data will be collected. Therefore, both the arm allocation, and the order in which clinics will have data collected are randomised.

### Power and sample size

Overall sample size: 1,200 (67 per cluster)

The impact evaluation has been powered to detect a difference in the primary outcome of “*correct SpO_2_ management*” pairwise comparison between the two new device arms (Acare LB-01 and Phefumla oximeters) and the control arm (CONTEC). Based on data from Malawi, we estimated that 55% of children within control clinics will meet the definition for correct SpO_2_ management (per primary definition 1). Using the following parameters, we will be able to detect a 22% increase in the primary outcome (primary definition 1) (i.e. to 77%): 18 months recruitment; 6 clusters per arm; 60 children per cluster; 2.5% alpha after applying the Bonferroni correction for pairwise comparisons; >80% power; ICC of 0.05, and a coefficient of variation of 0.2. This will give us a sample of 360 children per arm (n=1,080 children). We estimate recruitment of an average of 3-4 children each day (~18 months) from each facility (six per arm) yielding n=1,200 children when accounting for about 10% attrition (i.e., child enrolled but post-HCW visit examination incomplete). Given a lack of baseline data, we selected a relatively high ICC value and conservative coefficient of variation given the modest number of clusters.

### Primary outcome

‘**Correct SpO_2_ management**,’ a composite intermediate clinical endpoint between correct pulse oximeter use and hypoxemia outcomes that includes the three elements necessary to reduce inappropriately treated hypoxemia, and is defined as:

The proportion of children aged 0 to <24 months with acute respiratory infection and, (1) *device adoption*: HCW use of the device as evidenced by a HCW documented SpO_2_ and heart rate measured in room air (i.e., off of supplemental oxygen), (2) *quality SpO_2_* *measurement*: a study staff SpO_2_ measurement with the reference device that is within a 2% SpO_2_ range above or below the HCW documented SpO_2_, and (3) *correct SpO_2_* *decision-making*: an appropriate referral recommendation provided by the HCW according to the WHO-defined hypoxemia definition (SpO_2_ <90%).

**Table 1:** Key population and outcome definitions

| **Term** | **Definition** |
| --- | --- |
| Acute respiratory infection | Child aged 0 to <24 months with cough (reported or observed) or difficult breathing (reported or observed). |
| Pneumonia | Child aged 0 to <24 months with acute respiratory infection *plus* either fast breathing for age, chest wall indrawing, a general danger sign, or hypoxemia. |
| WHO-defined hypoxaemia | SpO_2_<90% while breathing in room air.^16^ |
| Study-defined hypoxaemia | SpO_2_<94% while breathing in room air^17^ |
| Moderate hypoxaemia | SpO_2_ 90-93% while breathing in room air |
| Biologically plausible | SpO_2_ measurements achieving a stable plethysmography waveform without any SpO_2_ change for 3-5 seconds and a heart rate between the 1^st^-99^th^ centile for age.^18^ |
| Appropriate referral | Children with an SpO_2_<90% have been counselled to go to the hospital and were given a pre-referral dose of antibiotic according to IMCI and oxygen (if available). Children with an SpO_2_>90% may also have been referred due to other clinical signs – this will not be considered inappropriate. |
| Difficult breathing | Any abnormal breathing pattern reported by caregiver or observed by HCW / study staff. |
| Fast breathing for age | Respiratory rate >60 breaths per minute if 0 to <2 months of age, >50 breaths if 2 to <12 months of age, or >40 breaths if 12 to <24 months of age. |
| Chest wall indrawing | Bilateral inward pulling of anterior chest wall subcostal tissue and lower ribs during inspiration. |
| General danger signs (2 to <24-month-olds) | According to WHO IMCI guidelines are any of: vomiting everything, lethargy or unconscious, unable to drink or breastfeed, convulsions, stridor at rest. |
| Neonatal danger signs (0 to <2-months-old) | According to WHO IMCI guidelines are any of: unable to feed well, not moving at all or moves only when stimulated, severe chest indrawing, grunting. |
| Successful reference SpO_2_ measurement | Within 10 minutes of the HCW measurement, study staff initiated the measurement and were able to obtain a biologically plausible measurement using the Masimo RadG reference device. |

### Secondary outcomes

- **Correct SpO_2_ management (definition 2):** The proportion of children aged 0 to <24 months with acute respiratory infection and (1) a HCW-documented SpO_2_ and heart rate measured in room air (i.e., off of supplemental oxygen), *and* (2) an appropriate referral recommendation has been provided by the HCW according to their WHO-defined hypoxaemia status (i.e. SpO_2_ <90% referred to hospital) *and* (3) SpO_2_ confirmed by a successful study staff measurement with the *device assigned to arm* within +/-2% of the HCW documented SpO_2_. *Note: Definition does not consider reference measure.*
- **Correct SpO_2_ management (definition 3)**: The proportion of children aged 0 to <24 months with acute respiratory infection and (1) a *HCW-documented* SpO_2_ and heart rate measured in room air (i.e., off of supplemental oxygen), *and* (2) an appropriate referral recommendation has been provided by the HCW according to their WHO-defined hypoxaemia status (i.e. SpO_2_ <90% referred to hospital) *and* (3) hypoxemia status confirmed by a successful study staff measurement with *reference device* (either hypoxemic or not hypoxemic). *Note: Definition does not require +/-2% difference with reference measure.*
- **Correct SpO_2_ management (definition 4):** The proportion of children aged 0 to <24 months with acute respiratory infection and (1) a *HCW-documented* SpO_2_ and heart rate measured in room air (i.e., off of supplemental oxygen), *and* (2) an appropriate referral recommendation has been provided by the HCW according to their WHO-defined hypoxaemia status (i.e. SpO_2_ <90% referred to hospital) *and* (3) hypoxemia status confirmed by a successful study staff measurement with *device assigned to arm* (either hypoxemic or not hypoxemic). *Note: Definition does not consider reference measure.*
- **Quality measurement frequency:** Proportion of biologically plausible measurements among all documented SpO_2_ measurements by study staff and HCWs using the assigned pulse oximeter device per arm and reference device.
- **Referral acceptance:** Amongst WHO-defined hypoxemic children, the proportion of children who present to a hospital within 24 hours.
- **Oxygen treatment:** Amongst WHO-defined hypoxemic children, the proportion who of children who present to a hospital and are given oxygen treatment within 24 hours.

## Aim 2: Methods, procedures and outcomes

Aim 2 will be addressed through a prospective observational cohort study nested within the cRCT (Figure 1). We will pool the 3 arms of the trial together and follow participants until 14-days post-enrolment. The primary descriptive outcome of interest is treatment failure (define under the *Primary Outcomes* section below), and the secondary descriptive outcome is hypoxemia prevalence. We will conduct pre-specified subgroup analyses by enrolment diagnosis, demographics, clinic type, clinic location, co-morbidities, and disease severity. We will classify children per IMCI pneumonia categories. We will also implement Aim 2 over an 18-month period.

### Participants

Same as under Aim 1.

### Power and sample size

The sample size for this nested aim was based on the power needed for the medical device evaluation (Aim 1), where we anticipate the enrolment of 1,200 children.

### Primary outcomes

Our primary descriptive outcome is treatment failure, defined as:

The proportion of enrolled children with a completed follow-up whose caregiver reported that they were still sick or have any of the following signs: cough at 14 days, difficult breathing at 14 days, change in antibiotic treatment or re-admission to clinic/hospital or death anytime at or before 14 days post study enrolment as confirmed by phone or home visit.

### Secondary outcomes

- **Hypoxemia prevalence (WHO-defined):** the proportion of all enrolled children who completed their intake/first (clinic) visit and had a successful study staff SpO_2_ measurement, with an SpO_2_<90%, using the reference device.
- **Hypoxemia prevalence (study defined):** the proportion of all enrolled children who completed their intake/first (clinic) visit and had a successful study staff SpO_2_ measurement, with an SpO_2_<94%, using the reference device.
- **Hypoxemia prevalence (moderate):** the proportion of all enrolled children who completed their intake/first (clinic) visit and had a successful study staff SpO_2_ measurement, with an SpO_2_ 90-93%, using the reference device.
- **Mortality:** measured among all enrolled children who successfully completed two-week follow up and defined as the number of children who have died from any cause within 14 days of recruitment.

# Statistical analyses

## Aim 1: General Statistical Analysis Principles

### Masking:

A final cleaned trial analysis dataset will be prepared by Carina King, with all the participant information removed and cluster allocation blinded. The trial statistician, Bareng Aletta Nonyane, will be provided with these data only to conduct the primary blinded trial analysis. Trial allocation will be revealed after the Expert Advisory Board has given permission to unblind the data. The remaining analyses will be done by Carina King, using the anonymised but unblinded dataset.

### Analysis population:

The primary analysis will be an *intention to treat analysis* (i.e., analysed according to intervention allocation rather than intervention receipt).

For all primary and secondary outcomes, we will conduct pairwise comparisons between the two new device arms (Acare LB-01 and Phefumla oximeters) and the control arm (CONTEC).

### Target primary estimand

We will estimate participant-average treatment effects for the primary and secondary outcomes of SpO_2_ management (See reference https://doi.org/10.1093/ije/dyac131) to make inference about the effect of using the new device compared to CONTEC in managing SpO_2_. We will assess the variability in cluster-sizes and whether these are associated with our target outcomes to apply appropriate models to obtain participant-average treatment effects.

### Covariate adjustment to improve precision in the estimates

Any covariates used for adjustment in the regression models will be those that are believed, from the literature or the team’s prior studies, to be associated with the outcomes. These may include participant sex, age, weight, general danger signs, signs of respiratory distress, comorbidities, and disease severity. Known clinic-level factors that may be associated with the outcome such as number of staff, infrastructure such as uninterrupted power supply, access to ambulance services will be described and assessed for imbalance between study arms. These covariates will be summarised using proportions and means between trial arms and reviewed by the study team and Expert Advisory Board. An unadjusted analysis will be primary while an adjusted analysis will be a sensitivity check and to improve the precision of the primary effect estimate.

### Interim analyses

An interim analysis is not planned.

### Missing data

We anticipate a low level of missing data, and therefore our primary analysis will be a complete cases analysis.

### Hypothesis tests and type 1 error rate

All hypothesis tests will be two sided with type 1 error rate of 0.05.

### Software

Stata Version 15 or later (Stata Corp, TX)

## Aim 1: Outcome definitions and analytic approaches

| **Table 2: Aim 1 Outcomes and Primary Analytic Approaches** | | |
| --- | --- | --- |
| **Outcome** | **Outcome definition** | **Analysis approach** |
| **PRIMARY**  The proportion of children aged 0 to <24 months with acute respiratory infection and, (1) *device adoption*: HCW use of the device as evidenced by a HCW documented SpO_2_ and heart rate measured in room air (i.e., off of supplemental oxygen), (2) *quality SpO_2_* *measurement*: a study staff SpO_2_ measurement with the reference device that is within a 2% SpO_2_ range above or below the HCW documented SpO_2_, and (3) *correct SpO_2_* *decision-making*: an appropriate referral recommendation provided by the HCW according to the WHO-defined hypoxemia definition (SpO_2_ <90%). | For each child with a successfully completed study staff measurement, a binary outcome (correct or incorrect management) will be generated. | We will conduct individual-level analyses to compare LB-01 or Phefumla oximeter to the control arm  *If cluster-size is not informative of the outcome:* mixed-effects logistic regression model, with the intervention treated as a fixed effect and cluster as a random effect. *If cluster-size is informative of the outcome: we will conduct a cluster-level weighted analysis with cluster sizes as weights with robust standard errors. Given the small number of clusters, a nonparametric cluster-level analysis will also be used as a secondary check* |
| **SECONDARY OUTCOMES** | | |
| **Correct SpO_2_ management** (definitions 2-4) | The same approach will be taken as the primary outcome. | The same approach will be taken as the primary outcome. |
| **Quality measurement frequency:** Proportion of biologically plausible measurements among all documented SpO_2_ measurements by study staff and HCWs using the assigned pulse oximeter device per arm and reference device. | For each child with a HCW documented SpO_2_ measurement, a binary outcome (biologically plausible measure) will be calculated  For each child with a successfully completed study staff measurement, a binary outcome (biologically plausible measure) will be calculated |  |
| **Referral acceptance:** Amongst WHO-defined hypoxemic children, the proportion of children who present to a hospital within 24 hours. | For each child with a HCW documented SpO_2_<90%, a binary outcome (presentation to hospital within 24 hours of recruitment) will be calculated |  |
| **Oxygen treatment:** Amongst WHO-defined hypoxemic children, the proportion who of children who present to a hospital and are given oxygen treatment within 24 hours. | For each child with a HCW documented SpO_2_<90%, a binary outcome (oxygen within the hospital given within 24 hours of recruitment) will be calculated |  |

#### Secondary analyses

- **Sub-group analyses:** The analyses in Table 2 will be repeated stratifying by the following covariates where feasible (i.e. sufficient sample size): child age (0 to <2 months, 2 to 11 months, 12 to <24 months) and sex; disease severity (presence of danger sign or not), clinic type (city or provincial).
- **Trends:** We will use graphical displays to study trends correct/incorrect management over time. Log-linear regression models will be used to quantify these trends and adjust for cluster-level heterogeneity over time.
- **Decomposed outcome:** We will conduct a secondary analysis which uses each component of the primary outcome definition as its own binary outcome (i.e. HCW documented SpO_2_, correct referral decision based on SpO_2_, HCW and study staff reference measure are within +/-2% of each other).

#### Sensitivity analyses

A sensitivity analysis will adjust for individual-level covariates that are associated with the outcomes (described above) and in consultation with the Expert Advisory Group.

## Aim 2: General Statistical Analysis Principles

### Missing data

We anticipate a low level of missing data, and therefore our primary analysis will be a complete cases analysis.

### Hypothesis tests and type 1 error rate

All hypothesis tests will be two sided with type 1 error rate of 0.05.

### *Software*

Stata Version 15 or later (Stata Corp, TX)

## Aim 2: Outcome definitions and analytic approaches

| **Table 3: Aim 2 Outcomes and Primary Analytic Approaches** | |
| --- | --- |
| **PRIMARY**  **Treatment failure:** The proportion of enrolled children who reported that they were still sick or whose caregiver reported any of the following signs: cough at 14 days, difficult breathing at 14 days, change in antibiotic treatment or re-admission to clinic/hospital or death anytime at or before 14 days post study enrolment as confirmed by phone or home visit. | **Outcome measurement**  Prevalence estimates of treatment failure, measured as a proportion based on the binary outcome (treatment failure, yes/no). |
|  | **Analytic approach**  This will be an exploratory analysis.  Logistic regression with a random effect for clusters (i.e. clinic) will be used to characterize the association between treatment failure and covariates of interest (pneumonia classification, age group, sex, hypoxaemia status, clinical presentation, comorbidities). We will calculate post-estimation probabilities (prevalence) of treatment failure by characteristic of interest.  **Secondary analysis**  We will explore an adjusted definition for treatment failure and its association with covariates of interest, excluding children who died from the analysis (and thereby from the primary outcome definition). We will also explore the relationship between treatment failure prevalence and HCW-documented SpO2 (criteria 1 for the primary outcome of the cRCT analysis) (yes/no) and our Aim 1 primary outcome of interest (correct management, yes/no). |
| **SECONDARY**  **Mortality:** among all enrolled children who successfully completed two-week follow up and defined as the number of children who have died from any cause within 14 days of recruitment. | **Outcome measurement**  We will summarize the number of children who have died as a rate (per 100 child participants) by covariates of interest (pneumonia classification, age group, sex, hypoxaemia status, clinical presentation, comorbidities). |
|  | **Analytic Approach**  A robust Poisson regression model with random effects for clusters (i.e. clinic) will be used to characterize the association between mortality and covariates of interest (unadjusted). We will consider (depending on the prevalence of the event) analyses to explore adjusted IRs risk factors, controlling for age, sex, hypoxemia status, general or neonatal danger signs, severe acute malnutrition, other comorbidities, trial arm, oxygen treatment, and hospitalization. For hypoxemia, three definitions will be considered: SpO_2_<90% (WHO-defined); SpO_2_<94% (study defined) and SpO_2_90-93% (moderate). We will explore the use of zero-inflated Poisson regressions with robust standard errors for clustering as well as bootstrapped standard errors (resampling techniques) and methods like RELATE for determining sets of clinics with common characteristics to replace the random effect for clusters if convergence issues arise related to rarity of events. |
| **SECONDARY**  **Hypoxemia prevalence**: among all enrolled children who completed their intake/first (clinic) visit and had a successful reference SpO_2_ measurement, those whose SpO_2_<90% (WHO-defined) as well as those <94% (study defined) and 90-93% (moderate) | **Outcome measurement**  For each child, a binary outcome (hypoxemia yes/no) will be recorded and summarized as proportions by category: SpO_2_<90% (WHO-defined); SpO_2_<94% (study defined) and SpO_2_90-93% (moderate) |
|  | **Analytic Approach**  We will report hypoxemia prevalence by SpO_2_ category.  Binary (for SpO_2_<90%, SpO_2_<94%) and multinomial (for SpO_2_ 90-93%) logistic regressions will be used to characterize the association between hypoxemia and covariates of interest (pneumonia classification, age group, sex, clinical presentation, comorbidities). |
